# Effects of the Traditional Chinese Medicine Tang Luo Ning on Intestinal Flora and Oxidative Stress in Diabetic Rats

**DOI:** 10.1155/2020/3452625

**Published:** 2020-10-29

**Authors:** Xiaoyi Wei, Wenjing Zong, Yanbin Gao, Siyang Peng, Ke Liu, Yalin Zheng

**Affiliations:** Beijing Key Lab of TCM Collateral Disease Theory Research, School of Traditional Chinese Medicine, Capital Medical University, Beijing 100069, China

## Abstract

**Objective:**

To determine the effects of TLN on glycolipid metabolism, oxidative stress, and intestinal flora in diabetic rat.

**Materials and Methods:**

Thirty-five male Sprague-Dawley (SD) rats (180–200 g) were divided into two groups. The normal group was fed a standard-chow diet, whereas, in the model group, diabetes was induced by intraperitoneal administration of streptozotocin (STZ) combined with a high-fat sucrose diet. Then, the model group was randomly allocated to four groups: DM (diabetes model) and TLNH (TLN high dose), TLNL (TLN low dose), and NAC (N-acetylcysteine). Rats in the TLNH, TLNL, and NAC groups were intragastrically administered TLN and NAC for 12 weeks. Subsequently, their weights, fasting glucose levels, serum lipids, serum insulin, serum ROS, and intestinal flora were determined.

**Results:**

The weight and intestinal flora abundance of the DM group were significantly lower than those of the normal group, whereas their total serum cholesterol (TC), low-density lipoprotein cholesterol (LDL-C), serum reactive oxygen species (ROS), and serum insulin (INS) levels were significantly higher than those of the normal group. TC and LDL-C levels in the TLNL group and DM group were similar, whereas FBG, INS, and ROS levels in the TLNL group were obviously lower than those in the DM group. Compared with the DM group, there was a significant increase in intestinal flora abundance in the TLNL group. At the phylum level, the ratio of Firmicutes to Bacteroidetes (core microbiota) varied in all groups. However, in the DM group, Firmicutes abundance decreased, whereas that of Bacteroidetes increased. An opposite trend was observed in the TLN-treated groups.

**Conclusions:**

TLN, which showed a dose-dependent therapeutic effect, can effectively decrease serum lipid, serum insulin, blood glucose, and serum ROS levels. It can also rebalance the ratio of Firmicutes to Bacteroidetes. Furthermore, the low-dose TLN treatment was most efficacious.

## 1. Introduction

Diabetes mellitus prevention and treatment has become a major public health concern [1]. Some studies have shown that intestinal flora can directly participate in many physiological processes such as immune regulation and lipid metabolism, closely related to metabolic conditions such as diabetes mellitus, insulin resistance, obesity, and fatty liver [2, 3]. Improving intestinal flora structure is emerging as a new treatment for diabetes mellitus. Chinese medicine, which can “strengthen the body and eliminate pathogens” by regulating probiotics and inhibiting pathogenic bacteria, can reportedly regulate intestinal microecological balance and improve physical function [4]. The traditional Chinese medicine Tang Luo Ning (TLN) is a clinical herbal prescription of Professor Yanbin Gao of Capital Medical University. Clinical and animal experiments had shown that TLN can effectively treat diabetic peripheral neuropathy; however, the efficacy mechanism of it is unclear. Animal experiments have shown that TLN can effectively reduce blood glucose levels in STZ-induced diabetic rats [5–7]. Based on previous research, this study explores the relationship between therapeutic mechanism of TLN and intestinal flora.

## 2. Materials and Methods

### 2.1. Animals

Thirty-five 180–200 g specific pathogen-free (SPF) male SD rats were purchased from Charles River Inc. (Vital River Ltd., Beijing, China) and raised in the SPF animal room at the Department of Laboratory Animal Sciences, Capital Medical University (Beijing, China). Room temperature and relative humidity were maintained at 22°C and 26 ± 2%, respectively. Illumination time was regulated according to the normal circadian rhythm.

### 2.2. Drugs and Reagents

TLN is composed of eight crude drug materials, including *Astragalus membranaceus* (15 g), *Salvia miltiorrhiza* (15 g), *Rhizoma cibotii* (15 g), *Achyranthes bidentata* (12 g), *Yuanhu* (10 g), *Papaya* (15 g), *Paeonia rubra* (12 g), and *Caulis* (15 g), which were all purchased from Anguo Medicine Market (Hebei Province, China), in conformity with the standards of the Chinese Pharmacopoeia. TLN decoction preparation and chemical composition determination were performed as previously described [8]. N-acetylcysteine (NAC) was purchased from G-CLONE Co., Ltd. (Beijing, China). Triglyceride, total cholesterol, high density/low density lipoprotein cholesterol kits were purchased from InTec Products, Inc. (Xiamen, China), ROS ELISA kit from Myhalic Biotechnology Co., Ltd. (Wuhan, China), and STZ from Sigma-Aldrich (Munich, Germany).

### 2.3. Instruments

The following instruments were used: Olympus AU480 automatic biochemical analyzer (Olympus Co., Japan), Sigma 3K15 centrifuge (Sigma-Aldrich Co., Germany), SpectraMax Plus 384 Full Wavelength Enzyme Marker (Molecular Devices Co., USA), and Roche ACCU-CHEK blood glucose meter (Roche, Switzerland).

### 2.4. Animals and Treatments

Thirty-five male SD rats were maintained on a standard-chow diet for 1 week and then blood samples collected from their tails were used to establish their fasting glucose and lipid levels. After screening out those with abnormal glucose and lipid levels, the rats were divided into the normal group (7 rats), which continued to receive the standard-chow diet and the model group (28 rats), which received a high-fat sucrose diet (HFSD), containing basic feed (58.8%), lard (10%), sucrose (20%), bile salt (0.2%), yolk powder (10%), and cholesterol (1%). After 4 weeks, all rats were fasted for 12 h and those in the model group were intraperitoneally injected 1% STZ formulated in citrate sodium buffer (dose, 35 mg/kg), whereas those in the normal group were injected an equal volume of saline. Fasting blood glucose (FBG) was determined after 1 week and the model was assumed to be established if FBG was higher than 16.7 mmol/L. The 28 rats of the established model group were then randomly divided into four groups of seven rats each: DM (diabetic model), TLNH (TLN high dose), TLNL (TLN low dose), and NAC (N-acetylcysteine) groups. For 12 weeks, the normal group was maintained on a standard-chow diet, whereas the other groups were fed a HFSD, as previously described [8].

All animal experiments were approved by the Institutional Animal Care and Use Committee of Department of Laboratory Animal Sciences, Capital Medical University (Animal Experimental Ethics number (AEEI-2017-019)).

The equivalent therapeutic dose for rats was calculated according to the human and rat body surface area conversion algorithm. The following doses were obtained: TLNH, 10.9 g/kg/d; TLNL, 5.45 g/kg/d; NAC, 0.07 g/kg/d. The normal and DM groups were obtained equal volume of ddH_2_O. All treatments were intragastrically administered for 12 weeks.

### 2.5. Sample Collection

Body weight and FBG were regularly determined every two weeks. After 12 weeks, the animals were fasted for 12 h. Two hours after blood was collected from their abdominal aortas, the samples were centrifuged at 3000 rpm for 15 min and the obtained sera were stored at −80°C until use. Cecum contents were collected and stored at −80°C in sterile cryopreservation tubes.

### 2.6. Analysis of Biomarkers

Total serum cholesterol (TC) and low density lipoprotein cholesterol (LDL-C) were measured using an automatic biochemical analyzer. FBG, serum insulin (INS), and serum ROS were measured using a Roche blood glucose analyzer, a radioimmunoassay, and an ELISA kit, respectively. Rat fecal sample DNA purity and concentration were determined using a NanoDrop2000 ultramicrospectrophotometer. DNA integrity was determined using agarose gel electrophoresis and microecological detection was performed when DNA purity was established.

### 2.7. Analysis of Intestinal Flora

Cecum contents weighed 150–200 mg. Firstly, the samples were melted on the ice and were thoroughly mixed and centrifuged. After an appropriate amount of samples was taken, the purity and concentration of their DNA were measured by NanoDrop2000 ultramicrospectrophotometer, while the DNA integrity was detected by agarose gel electrophoresis. The samples were then stored in a refrigerator at −80°C and microecological detection of cecum contents will proceed if the purity was qualified. The total number of sequences of each sample in the OTU abundance matrix was randomly sampled at different depths and the rarefaction curves were drawn according to the number of sequences extracted at each depth and the corresponding OTU number.

### 2.8. Statistical Analysis

Statistical analyses were performed using SPSS version 17.0 from IBM Corp. (Armonk, NY, USA). Data are presented as mean ± SD. Multiple independent samples were compared using one-way analysis of variance (ANOVA). When there was variance homogeneity, the least significant difference (LSD) test was performed; otherwise, Tambane's T2 post hoc test was performed. A *p* value <0.05 was considered statistically significant.

## 3. Results

### 3.1. General Condition of Rats

**Figure 1:**
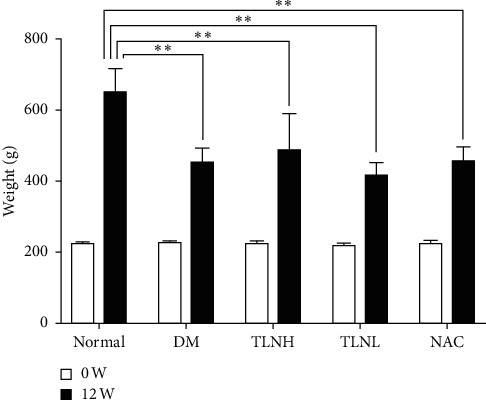
Comparison of weight of rats in each group. ^*∗∗*^*p* < 0.01 versus the normal group.

The normal group rats exhibited increased weight, bright fur, and good mental state, whereas the DM group rats presented yellow fur, soft stool, and a dispirited appearance. After STZ injection, the rats rapidly lost weight and slowly regained it. The weights of the DM group rats were significantly lower than those of the normal group rats (Figure 1).

### 3.2. Glucose and Lipid Metabolism of the Rats

TC and LDL-C levels in the DM group were significantly higher than those in the normal group (*p* < 0.01), were decreased in the TLN and NAC groups, and were significantly lower in the NAC group than in the DM group (*p* < 0.05).

The DM rats exhibited significantly higher INS levels than the normal group rats (*p* < 0.05). However, serum insulin levels decreased after TLN treatment. The improvement was more evident in TLNL group, which was significantly different from that of the DM group (*p* < 0.05).

**Figure 2:**
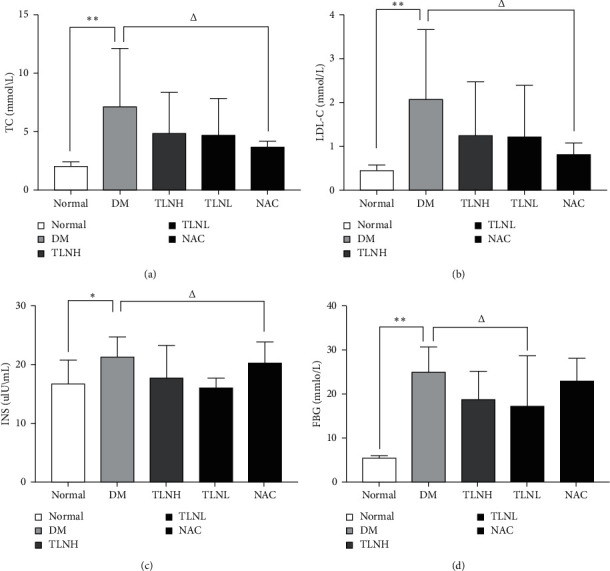
Effect on lipid and glucose metabolism under the treatment of TLN high dose (10.9 g/kg/d) and TLN low dose (5.45 g/kg/d). (a) Levels of TC and (b) the levels of LDL-C were detected by automatic biochemical analyzer. (c) INS levels were detected by radioimmunoassay. (d) FBG levels were detected by blood glucose meter. ^*∗*^*p* < 0.05  versus the normal group; ^*∗∗*^*p* < 0.01 versus the normal group; Δ*p* < 0.05 versus the DM group.

FBG levels in the normal group were significantly lower than those of the other groups (*p* < 0.01). The glucose level decrease in the TLNL group was most significant and this glucose level much lower than that in the DM group (*p* < 0.05) (Figure 2).

### 3.3. ROS Levels of the Rats

**Figure 3:**
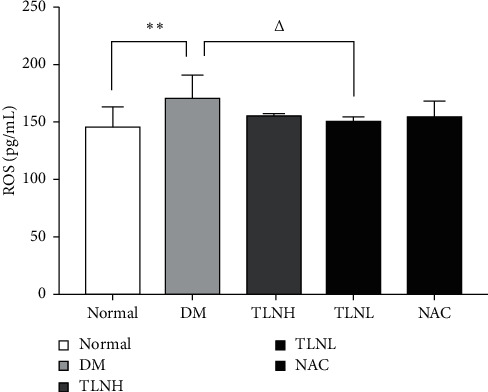
Effect of the TLN high dose (10.9 g/kg/d) and TLN low dose (5.45 g/kg/d) on ROS production. ^*∗∗*^*p* < 0.01 versus the normal group; Δ*p* < 0.05 versus the DM group.

ROS levels in the DM group were significantly higher than those in the normal group (*p* < 0.01) (Figure 3). In all treated groups, ROS levels decreased and those of the TLNL group were significantly lower than those of the DM group (*p* < 0.05).

### 3.4. Analysis of Intestinal Flora

**Figure 4:**
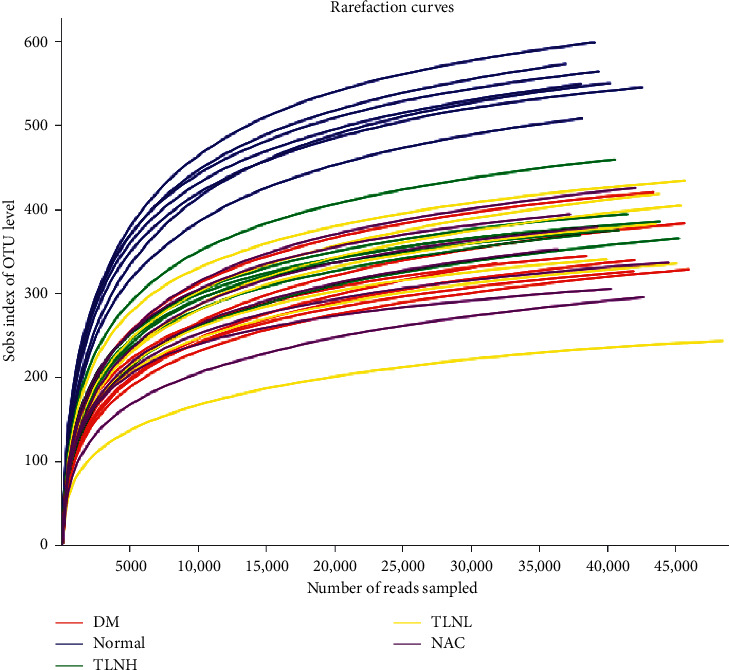
The horizontal axis represents the total number of sequences randomly selected in each sample. The vertical represents the number of OTU observed at the corresponding depth. After the total number of randomly selected sequences reached 25,000, the curves tended to flatten out, indicating that the sequencing result is enough to reflect the diversity contained in the current sample.

As shown in Figure 4, after the total number of randomly selected sequences reached 25,000, they tended to flatten out and the sequencing depth was qualified and could be further evaluated. After the detection of microbial DNA sequences in the samples, they were divided according to the database and the representative sequences of each OTU were used for classification status identification and phylogenetic analysis. According to the abundance distribution of OTU in different samples, the diversity of each sample was evaluated. Finally, the specific composition of each sample (among each group) at different classification levels was analyzed.

**Figure 5:**
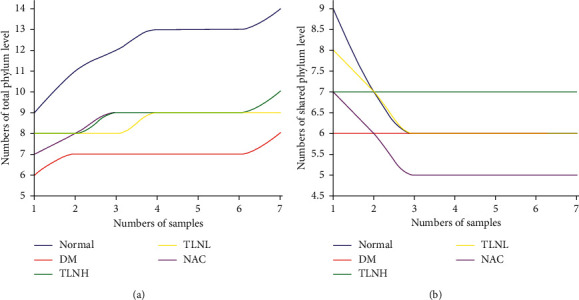
Pan/Core analysis. The horizontal axis represents the total number of sequences randomly selected in each sample. The vertical represents the number of OTU observed at the corresponding depth. (a) Pan analysis. (b) Core analysis.

Pan/Core species analysis is used to describe changes in the total number of species and core species as the sample size increases. It is widely used in biodiversity and community studies to determine whether the sample size is sufficient and to evaluate the total species richness and core species in the environment. Pan analysis showed that the total number of species in each group gradually increased with increasing sample numbers and that the number of core species in each group tended to stabilize with increasing sample size (Figure 5), suggesting sample size sufficiency in this experiment.

**Figure 6:**
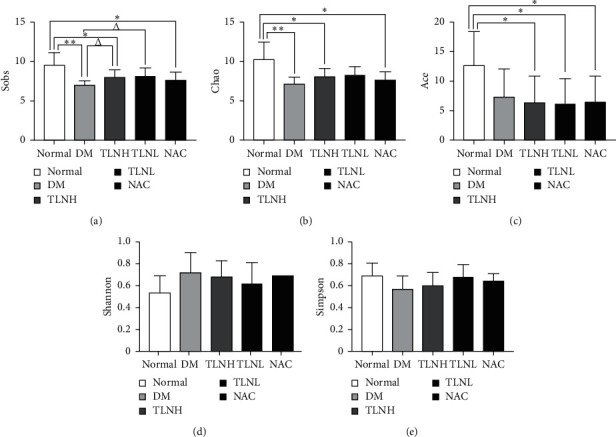
Diversity index. ^*∗∗*^*p* < 0.05 versus the normal group; ^*∗∗*^*p* < 0.01versus the normal group; Δ*p* < 0.05 versus the DM group.

Alpha diversity analysis was used to assess microbial community richness and diversity. Sobs, Chao, and Ace indexes are often used to describe community species richness. Shannon's and Simpson's indexes are used to characterize species diversity. Unlike the other four indexes mentioned above, Shannon index's value is negatively correlated with species diversity.  Figure 6 shows that DM group species richness was significantly (*p* < 0.01) lower than that of the normal group. Compared with the normal group, species richness was significantly lower in TLNH and NAC groups (*p* < 0.05). There was no significant difference in Sobs and Chao indexes between the normal and TLNL groups. Compared with the DM group, the Sobs index of the TLNH and TLNL groups was significantly higher (*p* < 0.05). The DM group's Simpson index was the lowest; however, there was no significant difference among the groups.

**Figure 7:**
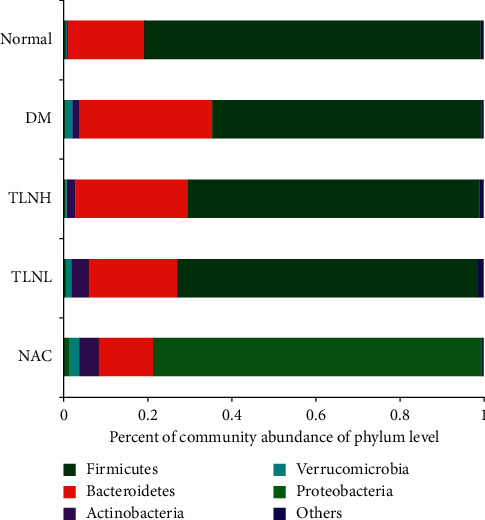
Community bar chart analysis. The horizontal axis represents the percent of community abundance on phylum level (%). The vertical represents the group name.

**Figure 8:**
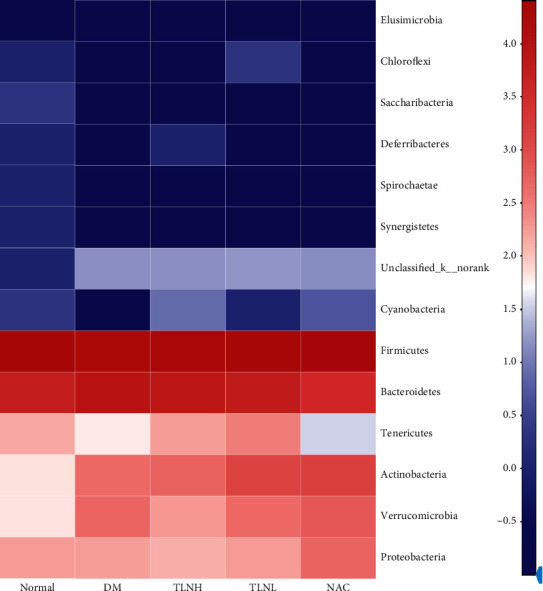
Community heatmap. The horizontal axis represents group names. The vertical axis represents the species name and the variation of abundance of different species in the sample is shown by color gradient of color block. The value represented by color gradient is on the right of the figure.

The community composition bar chart (Figure 7) and proportion (Table 1) show that the main phyla in each group were Firmicutes, Bacteroidetes, Actinobacteria, Verrucomicrobia, and Proteobacteria, while the core flora in each group were Firmicutes and Bacteroidetes. However, the ratio of the two different types of flora was significantly different. The community composition heatmap (Figure 8) presents information on community species composition by a color gradient, which reflects the similarity and difference in community composition in the different groups at different classification levels. The heatmap shows that the flora species in the normal group were more abundant than those of the other groups. In the DM group, Actinobacteria and Verrucomicrobia increased, whereas Tenericutes and Cyanobacteria decreased, compared with the normal group. In the NAC group, Proteobacteria increased compared with other groups, whereas Actinobacteria increased and Bacteroidetes decreased, compared with the DM group. Additionally, in the TLNH group, Tenericutes increased, whereas Verrucomicrobia and Proteobacteria decreased, compared with the DM group. Moreover, Tenericutes and Actinobacteria increased in the TLNL group compared with the DM group.

## 4. Discussion

Diabetes mellitus is a metabolic condition caused by dysfunction in insulin secretion. Its main symptom is hyperglycemia, accompanied by dyslipidemia and hypertension. Hereditary factors, high-fat and energy-dense diets, and a sedentary lifestyle are the major contributors to diabetes development. Diet is the major factor that induces intestinal microbiome changes.

The gastrointestinal tract is the habitat for trillions of microorganisms. The genome of the entire microbial community constitutes the intestinal microbiota. Studies have shown that, in the human growth and development process, intestinal bacteria taxonomy and function are dynamic and highly personalized and some species dominate at certain life stages [9]. Presently, the hypothesis of whether the intestinal flora of diabetic patients is characteristic or not has not been universally recognized in academic circles and, therefore, needs to be supported by more experimental data [10]. However, some studies have reported that an imbalance in the intestinal microbial ecology plays a role in the rapid progress of insulin resistance in type 2 diabetes mellitus. This imbalance can remodel intestinal barrier function, alter host metabolism and signal transduction pathways, and directly/indirectly relate to insulin resistance in type 2 diabetes mellitus [11, 12].

Our results indicated that the abundance of intestinal flora of diabetic rats with glucose and lipid metabolism disorder was reduced, the abundance of Firmicutes decreased, whereas Bacteroidetes increased, a trend that is supported by the findings of several clinical and experimental researches [13–16]. However, the abundance of intestinal flora of diabetic rats increased after TLN treatment; meanwhile, the relative abundance ratio of two dominant bacteria changed: Firmicutes increased while Bacteroidetes decreased. These variations demonstrated that the active components of TLN could be used as prebiotics to regulate the unbalanced intestinal flora, help restore the homeostasis of intestinal microbial system, and further affect the health of the host.

Oxidative stress is a risk factor in diabetes mellitus progression. Via various mechanisms, it can increase inflammatory cytokine production, which can affect intestinal nervous system function, causing intestinal motility disorders [17–19]. In our study, results showed that the changes of intestinal flora structure in the TLN treatment groups were consistent with the trend of improving glucose and lipid metabolism and reducing oxidative stress products. In addition, this effect is dose-dependent. This indicated that the mechanisms of TLN may adjust intestinal flora structure, reduce inflammation and oxidative stress, improve glucose and lipid metabolism, and, thus, contribute to reducing insulin resistance and treating diabetes. In addition, the regulating effect of TLN was closely related to its dose, which suggested that the relationship between dose and efficacy ought to be noticed in clinical use.

Our research team has for many years been devoted to research on the mechanism involved in the effect of TLN in diabetic peripheral neuropathy treatment. Accumulating evidence confirms that TLN has antioxidant and protective effects on sciatic nerve function and is a good prescription for the prevention and treatment of diabetic peripheral neuropathy [20–22]. The results of this experiment once again reflect the dual effect of TLN on antioxidant stress and glycolipid metabolism improvement. This study reveals that TLN's performance in intestinal flora structure regulation was highly consistent with its therapeutic effects, providing a powerful clue for further research on its blood sugar regulation mechanism and its protective effect on gastrointestinal nerves. Certain limitations exist in our study that the parameters were analyzed at a single time point when animals were sacrificed, together with the fact that the microbial agents were not added in the control group. In the follow-up study, the dynamics of parameters will be analyzed during the experiments. Thus, the dynamic changes of the correlation between each parameter and curative effect may be understood and the trend may be explored. Additionally, the experimental design will be further optimized to determine the specific pathway by which TLN regulates microbial flora and identify new potential therapeutic targets.

## 5. Conclusions

TLN effectively decreased hyperlipemia, hyperinsulinemia, and blood glucose in a dose-dependent manner; the effect observed in TLNL group was greater than that in TLNH group. TLN also effectively decreased ROS levels and oxidative stress; significant effects were observed in TLNL and NAC groups. It also rebalanced intestinal flora richness and proportion; TLN showed obvious therapeutic advantages in regulating the relative abundance ratio of the two dominant bacteria phyla: Firmicutes and Bacteroidetes.

## Figures and Tables

**Table 1:** Percent of community abundance on phylum level (%).

OTU ID	Normal	DM	TLNH	TLNL	NAC
Firmicutes	79.93	64.72	70.09	72.12	78.4
Bacteroidetes	18.56	31.4	26.41	20.4	12.88
Actinobacteria	0.2	1.48	1.81	4.28	4.64
Verrucomicrobia	0.21	1.61	0.6	1.55	2.22
Proteobacteria	0.58	0.52	0.41	0.57	1.68
Others	0.49	0.23	0.65	1.05	0.16

## Data Availability

The data used to support the findings of this study are available from the corresponding author upon request.
